# Effect of Systemic Antioxidant Allopurinol Therapy on Skin Flap Survival

**Published:** 2017-01

**Authors:** Mehdi Rasti Ardakani, Ahmed Al-Dam, Ashkan Rashad, Ali Shayesteh Moghadam

**Affiliations:** 1 Department of Plastic Surgery, Isfahan University of Medical Sciences, Isfahan, Iran;; 2 Department of Oral and Maxillofacial Surgery, University Medical Center Hamburg-Eppendorf, Hamburg, Germany

**Keywords:** Allopurinol, Antioxidant, Skin flap, Survival

## Abstract

**BACKGROUND:**

It has been reported that systemic administration of allopurinol improves cell survival. This study was aimed to evaluate effects of allopurinol on skin flaps in dogs.

**METHODS:**

Twenty dogs underwent one skin flap surgery with a 2-week interval. The first procedure was performed according to the standard protocols. The second phase was started by a 1-week pretreatment with allopurinol. Length of the necrotic zone was measured and recorded daily. At each phase, flaps were removed and sent for histopathological study after 1 week observation.

**RESULTS:**

Mean length of the necrotic zone in allopurinol treated skin flaps has been significantly less than normal flaps over all 7 days of observation (*p*<0.0001). Histopathology study showed less inflammation and more normal tissue structure in the allopurinol treated skin flaps.

**CONCLUSION:**

It was demonstrated that systemic administration of allopurinol significantly improved skin flap survival.

## INTRODUCTION

Skin flaps are increasingly used by plastic surgeons to reconstruct tissue defects resulting from trauma, ablative surgery or congenital malformation. Nevertheless, necrosis is still a serious complication which may affect skin flap survival.^1^^,^^2^ Despite remarkable progress in plastic surgery over the previous two decades, flap surgery is still associated with a notable morbidity. Total loss occurs in 1-5% of cases even in flaps which are microsurgically transferred by skilled surgeons.^3^^-^^5^ In addition, partial flap necrosis has been reported in 7-20% of free flaps and 20-33% of pedicled flaps.^6^^-^^9^

Post surgical ischemia-reperfusion (IR) injury is one of the most important causes of flap damage. IR injury may cause clinical problems for medical professions and prolonged hospitalization which increases medical costs.^10^^-^^12^ Neutrophil infiltration and production of superoxide free radicals are 2 major events which occur during IR, and lead to tissue injury.^13^^,^^14^ Xanthine oxidase (XO) plays an important role in the pathogenesis of IR injury. There is a significant up-regulation of XO system during ischemia of skin flaps.^15^^,^^16^

XO is a major source of reactive oxygen species (ROS) which triggers release of several inflammatory mediators.^17^^,^^18^ Allopurinol is an antioxidant drug, and inhibits XO. Hence, systemic administration of allopurinol reduces ROS formation and may improve survival rate of skin flaps.^19^^,^^20^ However, effectiveness of allopurinol in improving skin flap survival is still controversial.^21^^,^^22^ Combination of the above evidence and the necessity of finding an effective method to improve skin flap survival led us to design this study to evaluate effects of allopurinol on skin flaps in dogs.

## MATERIALS AND METHODS

This clinical trial was performed at veterinary hospital of our university medical centre. The study was approved by the local ethics committee (391136) and the guidelines of the Helsinki Declaration have been followed for this investigation. Twenty male, healthy, mix dogs aged between 3 to 4 years by the mean weight of 5.4 kg were included in the present study. All dogs had physical examination prior to the study. They had been completely vaccinated, and had received a complete course of anti parasite medication. All dogs were kept in separate cages during the study and were fed with normal diet.

In this study the dogs were divided to two groups of ten (Group A and B). All dogs underwent one skin flap surgery by a single blinded surgeon (a total number of 20 skin flaps). Group A (control group) procedure was performed under the standard conditions with no additional medication. Group B procedure was performed similar to the first one, but all dogs were pre-treated with unlabeled use of oral allopurinol from one week before the surgery until 48 hours after skin flap surgery. 

**Fig. 1:**
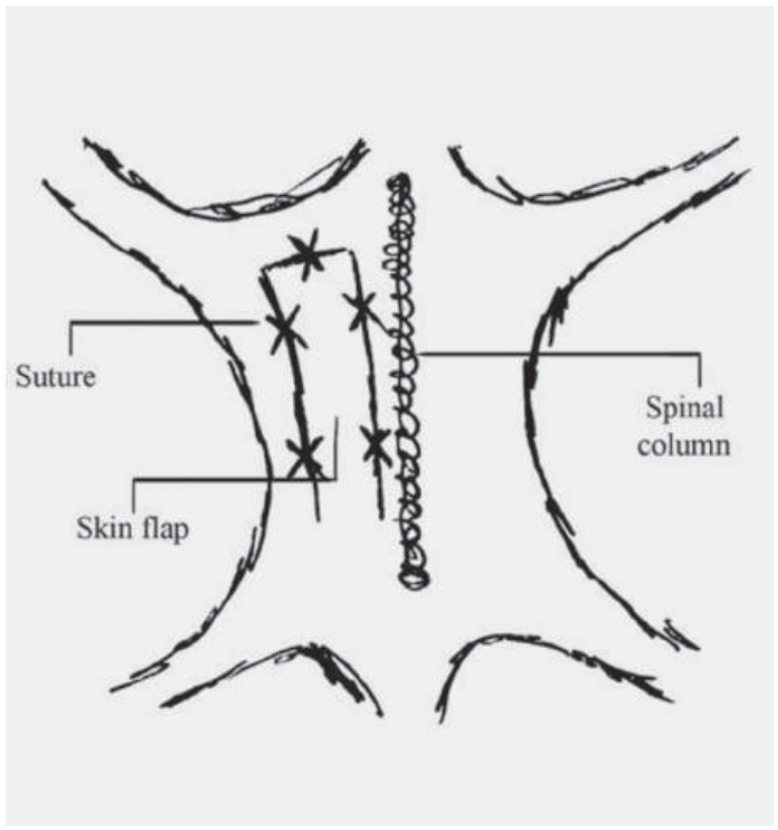
Schematic view of the surgical site and sutures’ sites.

In Group A as skin flap surgery group, after 12 hours of fasting, the surgical site (the back of the dogs) was shaved. Dogs were anaesthetized with ketamine (75 mg/kg of body weight) and a cuffed endotracheal tube was inserted into the trachea. Then an island adipofascial cutaneous flap, 4×24 cm, was created in one side of the spinal column. Afterwards, the flap was sutured in place immediately using simple interrupted nylon-01 sutures (Figure 1).

After the operation dogs were treated with appropriate dose of analgetics. In addition, 60 min before surgery and a daily dose of 30 mg/kg Cefazolin after surgery was commenced intravenously. All dogs were being visited over the first week after the operation on a daily basis by a veterinarian and the surgeon to check the general condition and skin flap status. At the end of this one week observation flaps were removed. After removal, flap site was closed with simple continuous suture of nylon-01. 

In Group B as skin flap surgery group, all ten dogs in second group received allopurinol (MD-Allopurinol ® 100 mg, Mehr Darou, Tehran, Iran, daily dose of 50 mg/kg of body weight) through the nasogastric tube for 9 days (7 days prior to surgery, 2 days during the observation). Skin flap surgery was performed one week after commencing allopurinol. Otherwise, the second operation was completely similar to the first group one. 

Regarding measurements, all flaps were assessed daily regarding the size of the necrotic area. After allowing the skin to lie in the natural position, length of the necrotic area was measured by the surgeon, using a single standard digital ruler with the accuracy of 1/100 centimeter. All measurements were recorded in cm. Length of the necrotic zone was defined as the longest distance between the necrotic edge and the survived area. Care was taken to ensure maintenance of a straight line during the measurement.

For pathologic study of the skin flaps, one week after each skin flap surgery, the skin flaps were removed, fixed in 10% formalin and sent for histopathological study. After hematoxylin and eosin (H&E) and toluidine blue staining, all samples were investigated by a single pathologist who was unaware of the type of treatment. Samples were studied regarding the number of neutrophils, fibroblasts, mast cells and other inflammatory cells, using a light microscope. Data was analyzed by SPSS 16.5 software. Independent-t test was used to compare mean length of necrotic zone between standard flaps and allopurinol treated flaps. P-values less than 0.05 were considered as the level of significance.

## RESULTS

**Fig. 2:**
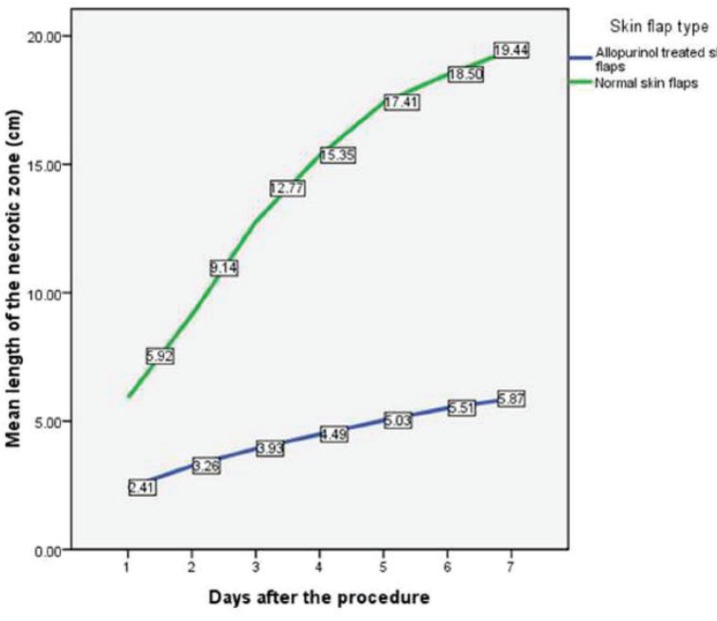
Illustration of changes in the mean length of necrotic zone in both groups. Data are presented as mean length of necrotic zone in cm in different days

**Fig. 3:**
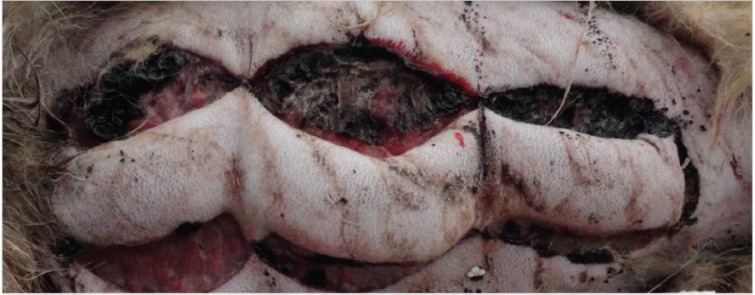
Postoperative view on allopurinol treated dog without necrotic area

**Fig. 4:**
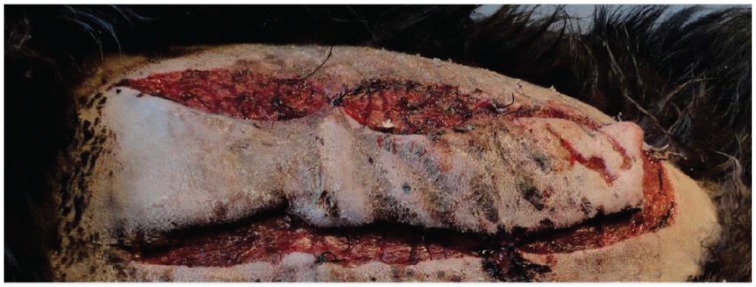
Vital flap mainly supplied by the basis allocated in allopurinol treated group

**Fig. 5:**
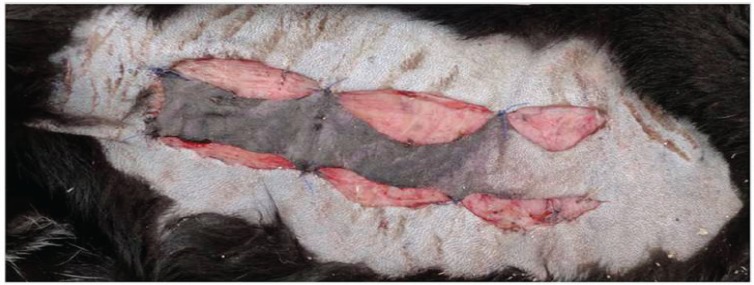
About two thirds of the base distant flap outlined to be necrotic without any allopurinol administration

**Fig. 6:**
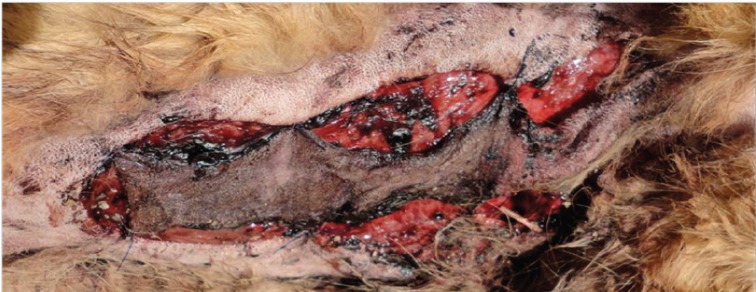
Insufficiently perfused flap of control group without any medication treatment

Comparison of two types of skin flaps showed that mean length of the necrotic zone in allopurinol treated skin flaps have been significantly less than normal flaps over all 7 days of observation (Table 1). Figure 2 illustrates the changes of necrotic zone for both groups during observation time. Differences in length of necrotic zones even increased with continually growing observation time. Figure 3 and 4 represent skin flaps treated with allopurinol whereas Figure 5 and 6 show control group flaps.

**Table 1:** Comparison of mean length of necrotic zone between two types of flaps in different days

	**Mean length of the necrotic zone (cm)**						
	**Day 1**	**Day 2**	**Day 3**	**Day 4**	**Day 5**	**Day 6**	**Day 7**
Normal skin flaps (n=10)	5.92±0.66	9.14±0.75	12.77±0.56	15.35±0.63	17.41±0.75	18.50±0.80	19.44±0.77
Allopurino l treated skin flaps (n=10)	2.41±0.33	3.26±0.42	3.93±0.54	4.49±0.62	5.03±0.67	5.51±0.67	5.87±0.63
*p *value	<0.0001	<0.0001	<0.0001	<0.0001	<0.0001	<0.0001	<0.0001

Data are presented as mean±SD length of the necrotic zone, in cm, during the observation time. N=number of cases; SD=Standard deviation

**Fig. 7:**
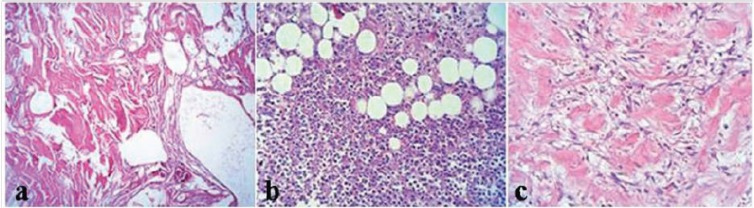
Histopathological findings of different zones of normal skin flap 7 days after skin flap surgery. A: necrotic zone, B: transitional zone, C: intact zone.

Regarding normal skin flaps findings of different zones, (i) necrotic zone findings: Coagulative necrosis with large amounts of vessel thrombosis can be detected. A few neutrophils and fibrotic tissue in margins were seen (Figure 7a). (ii) Transitional zone findings: Destruction of the epidermis in most of the regions. In superficial dermis there was infiltration of inflammatory cells- mostly neutrophils- between collagen bundles. Existing microabscess. More severe inflammation was found in the subcutis fat in form of non-septal panniculitis. Fat necrosis with intensive infiltration of inflammatory cells - neutrophils, macrophage and lymphoplasma cells could be detected. Numerous dilated vessels with evidence of thrombosis in some of them were present (Figure 7b). (iii) Intact zone findings: Moderate infiltration of inflammatory cells- macrophage and plasma cells - in the subcutaneous fat and deep dermis. There was focal fat necrosis. Furthermore existing extravasation of red blood cells and vasodilatation (Figure 7c). 

**Fig. 8:**
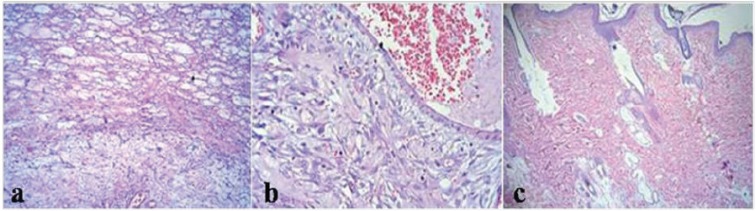
Histopathological findings of different zones of allopurinol treated skin flap 7 days after skin flap surgery. A: necrotic zone, B: transitional zone, C: intact zone

Regarding allopurinol treated skin flaps findings of different zones, (i) Necrotic zone findings: Shedding of the superficial epidermis. Intensive infiltration of inflammatory cells - neutrophils and macrophage - around the muscle bundles with focal microabscess and dilated vessels and thrombosis in some of them (Figure 8a). (ii) Transitional zone findings: Focal shedding of the epidermis accompanied by mild infiltration of neutrophils. Obvious red blood cell extravasation in superficial dermis. Severe panniculitis, fat necrosis, infiltration of neutrophils and dilated vessels with some thrombosis were seen in the deep dermis (Figure 8b). (iii) Intact zone findings: Completely normal structure of the skin tissue (Figure 8c).

## DISCUSSION

Despite several treatment strategies which aim at reducing or preventing skin flap necrosis, improvement of the flap survival is still an important concern for surgeons. This study showed that pre-/ and post-treatment with allopurinol leads to significant reduction of the necrotic zone length, more normal histological structure and less inflammation of the skin tissue in dogs. Allopurinol has been primarily used for treatment of hyperuricemia and gout,^23^ but based on its mechanism of action and its effects on endothelial function, it has been investigated as a novel medication for other medical problems.^24^^-^^27^ Effects of allopurinol on survival of skin flaps are related to its mechanism of action and mechanisms which cause tissue damage. IR injury has been considered as one of the major causes of skin flap damage.^28^

ROS including oxygen ions, free radicals and peroxides are generated during ischemic phase and especially during reperfusion. The production ROS over the reperfusion phase results in endothelial cell swelling, vasoconstriction and increased capillary permeability which impairs the microcirculation.^20^^,^^29^ ROS are usually produced by two major mechanisms: The Nicotinamide Adenine Dinucleotide Phosphate (NADPH) oxidase system in neutrophils and the XO system in endothelium cells.^12^^,^^30^^,^^31^ The XO system appears to be a major source of ROS.^12^^,^^32^^-^^34^ The activity of this system significantly increases during the ischemia, and produces a large amount of ROS.^20^ Therefore, it is not surprising to find that systemic administration of an XO inhibitor such as allopurinol improves skin flap survival.

A previous study performed by Tamir *et al.* confirms our findings by a different study design in rats. They investigated effects of allopurinol on survival of island skin grafts under prolonged period of ischemia and found it a useful method to improve skin flap survival.^28^ They believe that pre-treatment with allopurinol enables skin grafts to tolerate longer periods of ischemia. This effect is contributed to the XO inhibitor properties of allopurinol. Another study by Im *et al.* also reported that allopurinol can improve skin flap survival in rats by inhibiting most of the increased activity of XO during IR, and consequently, by preventing tissue damage.^35^

Picard-Ami *et al.* reported different levels of XO activity in rats, pigs and humans.^36^ Therefore, in contrast to the above mentioned studies, they studied effects of allopurinol on skin flaps of pigs and found it ineffective.^22^ This finding shows that although allopurinol can improve skin flap survival by reducing ROS levels, this effect is completely dependent on the level of XO activity in the target organ. Even though critical flap monitoring is within 72 hours postoperatively,^37^ we did monitor the flaps over 1 week. Nevertheless our study lacks an extended observation period and thus no statement can be made regarding long-term survival.

The present experiment had some critical differences with aforementioned studies. Instead of rats which are most commonly used to investigate this subject, we used dogs. Accordingly, flaps could be dimensioned generously comparable to humans with no size limitation as for rats. More importantly, in contrast to the previous studies in which allopurinol was administrated a few hours before^22^ to or even after the procedure,^28^ in this study dogs were pre-treated with allopurinol for 1 week prior to the surgery which helped us to reach a steady plasma concentration of the drug. 

In summary, findings obtained from our study demonstrated that systemic administration of allopurinol significantly improved skin flap survival and had a protective effect against flap necrosis in dogs. Clinical impressions were confirmed by histopathological examination. Although results of this method regarding administration of allopurinol are promising, further studies - such as comparison of XO activity of dogs with humans - are required prior to apply it on humans. Furthermore, studies with even smaller and larger dimensioned flaps should be done. Unfortunately, there is no uniform and practical way for worldwide use until now. Therefore further studies need to be done to reach a common method for make it practical.
